# Sigma-1 receptor agonist fluvoxamine for delirium in patients with Alzheimer's disease

**DOI:** 10.1186/1744-859X-9-6

**Published:** 2010-01-20

**Authors:** Tsutomu Furuse, Kenji Hashimoto

**Affiliations:** 1 Department of Psychiatry, Asahikawa Red Cross Hospital, Asahikawa, Japan; 2 Division of Clinical Neuroscience, Chiba University Center for Forensic Mental Health, Chiba, Japan

## Abstract

**Background:**

Delirium in older adults is a common and serious acute neuropsychiatric syndrome, with core features of inattention and global cognitive impairment. Although antipsychotic drugs are the medications most frequently used to treat this syndrome, these drugs are associated with a variety of adverse events, including sedation, extrapyramidal side effects, and cardiac arrhythmias.

**Methods:**

We report on two cases in which monotherapy of the selective serotonin reuptake inhibitor and sigma-1 receptor agonist fluvoxamine was effective in ameliorating the delirium of patients with Alzheimer's disease.

**Results:**

Delirium Rating Scale (DRS) scores in the two patients with Alzheimer's disease decreased after fluvoxamine monotherapy.

**Conclusion:**

Doctors should consider that fluvoxamine could be an alternative approach in treating delirium in patients with Alzheimer's disease because of the risk of extrapyramidal side effects by antipsychotic drugs.

## Background

Delirium in older adults is a common and serious acute neuropsychiatric syndrome, with core features of inattention and global cognitive impairment [1]. Antipsychotic drugs are the medications most frequently used to treat this syndrome, although exposure to these drugs can itself pose a risk for the subsequent development of delirium. Furthermore, antipsychotic drugs are associated with a variety of adverse events, including sedation, extrapyramidal side effects, and cardiac arrhythmias. Although the pathophysiology of delirium is not fully understood, current evidence suggests that drug toxicity, inflammation and acute stress responses can all contribute to a disruption of neurotransmission (for example, acetylcholine, glutamate, γ-aminobutyric acid, dopamine, serotonin, norepinephrine) and, ultimately, to the development of delirium [1].

The endoplasmic reticulum protein sigma-1 receptors play a key role in Ca^2+ ^signalling and cell survival, and have been shown to regulate a number of neurotransmitter systems in the brain [2-6]. The selective serotonin reuptake inhibitor (SSRI) fluvoxamine is a very potent agonist at sigma-1 receptors, which are also implicated in cognition and the pathophysiology of neuropsychiatric diseases [2-6]. A study using the selective sigma-1 receptor agonist [^11^C]-SA4503 and positron emission tomography demonstrated that fluvoxamine binds to sigma-1 receptors in living human brain at therapeutic doses, suggesting that sigma-1 receptors might play a role in the mechanism of action of fluvoxamine [7].

Given the role of sigma-1 receptors in the regulation of neurotransmitter systems, we hypothesised that fluvoxamine might be effective in the treatment of delirium. Here we report two cases in which fluvoxamine was effective in ameliorating the delirium of patients with Alzheimer's disease.

## Case reports

### Case 1

The patient was an 82-year-old Japanese woman who was diagnosed with Alzheimer's disease according to the Diagnostic and Statistical Manual of Mental Disorders, fourth edition (DSM-IV) and International Classification of Diseases, 10th edition (ICD-10) criteria. Brain computed tomography (CT), magnetic resonance imaging (MRI), and single photon emission computed tomography (SPECT) were also performed. Brain CT showed brain atrophy and ventricular enlargement, and MRI showed small infarcts in the brain. *N*-isopropyl- [^123^I]*p*-iodoamphetamine ([^123^I]-IMP)-SPECT showed the reduction of blood flow in the posterior cingulate cortex and lateral occipital cortex. Since she has hypertension and diabetes, antidiabetic and antihypertension treatments were administered before the development of delirium. She was hospitalised due to lung congestion that was detected by chest radiography. Her sleep disturbance was not improved by benzodiazepines, and she developed visual hallucinations of something. A psychiatric consultation was scheduled, and revealed disorientation and memory deficits. Her Delirium Rating Scale (DRS) [8] and Mini-Mental Scale Examination (MMSE) [9] scores were 17/32 and 20/30, respectively. Treatment with fluvoxamine (25 mg) was initiated after dinner, and next day increased to 50 mg. At 2 days after beginning treatment with fluvoxamine, her DRS score had decreased to 5/32, and both her delirium and sleep disturbance improved.

### Case 2

The patient was a 77-year-old Japanese woman who had been diagnosed with Alzheimer's disease according to the DSM-IV and ICD-10 criteria. Brain CT, MRI, and SPECT were also performed. Before the development of delirium, she had been treated with olanzapine (5 mg) because of her disorientation. She was hospitalised due to her persecutory delusions. At the time of hospitalisation, her DRS and MMSE scores were 17/32 and 21/30, respectively. Treatment with fluvoxamine (50 mg, twice a day) was initiated, and the next day increased to 100 mg since there were no gastrointestinal side effects. Her tendency to reject medication gradually improved 3 days after beginning treatment with fluvoxamine. At 1 week later, her DRS score had decreased to 8/32, and her condition is currently stable.

## Discussion

To our knowledge, this is the first report demonstrating that fluvoxamine monotherapy is effective for treating the delirium of patients with Alzheimer's disease. Nonetheless, a randomised double-blind, placebo-controlled study of fluvoxamine will be needed to confirm its efficacy for the treatment of this syndrome. In addition, it is currently unclear whether sigma-1 receptors are involved in the action of fluvoxamine on delirium. In order to confirm the role of sigma-1 receptors in the treatment of delirium, a randomised double-blind, placebo-controlled study of the selective sigma-1 receptor agonists (for example, cutamesine (SA4503)) in patients with delirium would also be of interest.

Previously, it has been reported that the combination of SSRIs with antipsychotic drug(s) and concomitant benztropine might increase the risk of delirium in patients [10-13]. Byerly *et al*. [12] reported a case showing delirium associated with sertraline, haloperidol and benzotropine. Furthermore, Armstrong *et al*. [13] reported a case of delirium in a patient who was taking benztropine and paroxetine concomitantly. These authors suggest that the addition of sertraline or paroxetine may cause a clinically meaningful inhibition of benztropine metabolism or an inhibition of central cholinergic function [12,13]. Nonetheless, the precise mechanisms underlying the incidence of delirium associated with the combination of sertraline (or paroxetine) and benztropine are currently unclear. Recent findings suggest that sigma-1 receptors might be involved in the different mechanisms of some SSRIs [4]. Fluvoxamine is a potent sigma-1 receptor agonist, and sertraline may be a sigma-1 receptor antagonist [4-6,14-16]. Paroxetine is a weak at sigma-1 receptors [4]. Taken together, it is likely that the difference for pharmacological actions (agonist or antagonist) of SSRIs at sigma-1 receptors may be involved in the mechanisms of different effects of these SSRIs [4-6] although a further detailed study is necessary.

Delirium is regarded as syndrome that consists of several domains of symptoms, such as disturbance of consciousness, cognitions, and perceptions [17]. At present, it is unclear whether fluvoxamine monotherapy is effective for certain domain of delirious symptoms or for all symptoms equally. Given the role of sigma-1 receptors in the cognition [4-6], it seems that improvement of cognitive impairments by sigma-1 receptor agonist may be involved in the mechanisms of this drug although a further study will be necessary.

A previous meta-analysis of randomised placebo-controlled trials demonstrated an elevated risk of mortality in older patients with dementia who were treated with atypical antipsychotics [18]. This paper suggests that the widespread use of atypical antipsychotic drugs in older adults should be re-evaluated, since older patients with delirium may have dementia. Therefore, the sigma-1 receptor agonist fluvoxamine may serve as an alternative treatment option for older adults with delirium.

## Conclusions

These two cases suggest that fluvoxamine could be an alternative approach in treating delirium of patients with Alzheimer's disease because of the risk of extrapyramidal side effects by antipsychotic drugs. More detailed double-blind studies should be performed to clarify the role of sigma-1 receptors in the efficacy of fluvoxamine for delirium.

## Consent

Written informed consent was obtained from the all patients in this case report.

## Competing interests

The authors declare that they have no competing interests.

## Authors' contributions

TF contributed to the clinical and rating evaluations during the follow-up periods. KH conceived of the study and participated in its study and coordination. Both authors read and approved the final manuscript.
